# Concurrence of Inappropriate Antidiuretic Hormone Secretion and Cerebral Salt Wasting Syndromes after Traumatic Brain Injury

**DOI:** 10.3389/fnins.2017.00499

**Published:** 2017-09-06

**Authors:** Bo Shen, Lin Li, Ting Li

**Affiliations:** ^1^Department of Neurosurgery, The People's Hospital of Shanxi Province Taiyuan, China; ^2^Division of Neurocritical Care, The People's Hospital of Shanxi Province Taiyuan, China; ^3^Academic Department of Neurosurgery, Shanxi Medical University Taiyuan, China; ^4^Department of Clinical Nursing, The People's Hospital of Shanxi province Taiyuan, China

**Keywords:** the syndrome of inappropriate antidiuretic hormone, cerebral salt wasting syndrome, traumatic brain injury, hyponatremia, central venous pressure

## Abstract

Syndrome of inappropriate antidiuretic hormone (SIADH) and cerebral salt wasting syndrome (CSWS) as the two most common neuroendocrine diseases, have been recognized and understood by many neurologists. Although SIADH and CSWS are the common causes of central hyponatremia after traumatic brain injury (TBI), a few cases are mixed, with the coexistence of the two pathological pathomechanism. However, the mixed type of both SIADH and CSWS has not been clearly reported in any literature. Here, we present the first description of the concurrent syndrome of SIADH and CSWS after TBI in four patients who underwent standard diagnostic procedures, treatment and follow up. Our findings further support that this rare neuroendocrine disease may exist in clinical practice, in which the traditional-conventional treatment shows poor efficacy.

## Introduction

Brain contusion and brain swelling caused by TBI interfere with and disrupt the normal neuroendocrine function of the hypothalamus and pituitary system, thus forming the SIADH or CSWS and triggering central hyponatremia, of which the pathophysiology had been widely recognized and understood by neurologists (Moro et al., 2007; Hannon et al., 2012; Verbalis et al., 2013; Taylor et al., 2017). But differentiation between these two syndromes is difficult because of overlapping signs, symptoms, and specially laboratory data. Today in the clinical community, distinction between these is still based on patient's volume status (Kim and Joo, 2009). However, the pathological changes including diffuse brain swelling, traumatic subarachnoid hemorrhage, and intracranial hypertension due to severe TBI, are completely different from the spontaneous subarachnoid hemorrhage, sellar tumors, and other diseases, because the hypothalamus—pituitary system diffuse injury may sometimes cause a variety of neuroendocrine pathological changes occur simultaneously, resulting in some very complex diseases (Agha et al., 2004). These are probably the most likely etiological explanations for this particular neuroendocrine disease of concurrent syndrome of SIADH and CSWS. And for this extremely special and rare syndrome, the differential diagnosis is rarely objective if simply through the volume status.

To date, this concurrent syndrome has never been definitively reported in any literature. In fact, even if the content of the literature was linked to it occasionally, its assertions were often ambiguous (Kim and Joo, 2009; Hannon et al., 2012). It is for these reasons, neurologists usually do not consider it when dealing with refractory hyponatremia. Neurologists in the diagnosis of central hyponatremia usually only consider the above two syndromes, if not A that is B, and will not consider the possibility of simultaneous occurrence of the two syndromes. Furthermore, the traditional approach to the differential diagnosis by detecting central venous pressure (CVP) can provide little help in the diagnosis of this euvolemic hyponatremia. If the neurologist cannot make a correct diagnosis in the early stages, but blindly follow the simple SIADH or CSWS treatment principles to guide therapeutic protocols, will eventually result in disastrous consequences such as life-threatening hypovolemia or congestive heart failure and many more.

Four patients after TBI were diagnosed and treated as mixed types of the above two syndromes based on relevant clinical and laboratory examinations and treatments attempted between 2005 and 2016.The pathological features and the processes of onset, diagnosis, and treatment of all four cases were quite similar, they are reported as follows to further clarify the clinical characteristics, diagnosis basis, and treatment strategy of this rare disease.

## Background

### Patients reports

The patients were admitted 2–10 h after injury, Table 1 shows the diagnosis at admission for all patients as well as the Glasgow Coma Scores (GCS) post-admission but before sedation. All patients were healthy, with no previous medical history of diseases in the heart, lung, kidney, thyroid, and adrenal gland systems. After admission, epidural hematoma evacuation was performed for case 3, a left-sided craniotomy for epidural hematoma evacuation, and a right-sided decompressive craniotomy were performed for case 2, and tracheotomy was successively except for case 3.

**Table 1:** Clinical characteristics of four patients.

**Case number**	**Age**	**GCS on admission**	**Cause of TBI**	**Diagnosis**	**GOS after 6 months**
1	53	9	Collision	Diffuse axonal injury; SAH	5
2	65	6	Falling	Extradural hematoma; SAH	3
				Brain contusion	
				Diffuse brain swelling	
3	31	11	Traffic accident	Extradural hematoma; SAH	5
				Bifrontal contusion	
4	41	5	Traffic accident	Diffuse axonal injury; SAH	1
				Diffuse brain swelling	

### Clinical and laboratory testing

Daily input and output volume within 24-h, serum sodium level, plasma, and urine osmolality, urine specific gravity, 24-h total urinary sodium excretion were recorded. The related hormones, including thyroid hormone, cortisol, and adrenocorticotropic hormone, were tested every other day for all cases. The results of these were always normal; thus, the possibility that hyponatremia was caused by other endocrine diseases was excluded accordingly. For brain natriuretic peptide (BNP) measurements, the chemoluminescence method was utilized, with a cutoff value of 100 pg/ml to suggest abnormal. As to arginine vasopressin (AVP) measurements, the radioimmunoassay was applied, with the normal range 0.4–2.4 pg/ml(osm<285mosm/kg) and 2–12 pg/ml(osm>285 mosm/kg). Hemodynamic monitoring included non-invasive measurement of blood pressure and cardiac frequency. CVP was evaluated accurately from open-ended peripherally inserted central venous catheters (PICCs) using PHILIPS IntelliVue MP60.

## Results

Severe hyponatremia successively occurred in all patients, with the serum sodium extreme even lower than 125 mmol/l. The plasma osmolality was lower than the urine osmolality for all patients, as shown in Table 2. The parameters of the monitoring instruments before and after the onset of hyponatremia showed stable blood pressure and cardiac frequency, with no signs of negative fluid balance, such as decreased blood pressure and increased cardiac frequency. Serum BNP and AVP concentrations were significantly higher than before, and the CVP of all patients were in the normal range (Table 2). All patients were bedridden due to illness; therefore, their body weights could not be accurately measured. Following the principle of SIADH diagnosis and treatment (Verbalis et al., 2013), fluid restriction was provided. To avoid cerebral vasospasm, which is commonly complicated in the fluid restriction for neurosurgery patients, in accordance with the relevant literature (Musch and Decaux, 1998), the daily total liquid volume was adjusted to 2,000 ml/day. At the same time, for extreme situations in which the serum sodium were lower than 125 mmol/l, the infusion of 3% sodium chloride solution was provided accordingly (Ellison and Berl, 2007; Verbalis et al., 2013; Thongrong et al., 2014).

**Table 2:** Clinical parameters of hyponatremia in four patients.

**Parameters**	**Case 1**	**Case 2**	**Case 3**	**Case 4**
Time to hyponatremia (days)	13	12	10	9
Lowest serum sodium (mmol/l)	121	125	128	116
Urine flow rate (ml/d)	2,530	2,385	2,740	2,810
Urine specific gravity after hyponatremia	1.082	1.073	1.061	1.096
Serum/urine osmolarity (mosm/kgH_2_O)	269/940	264/970	278/750	273/890
Average 24-h urine sodium excretion (mmol)	869	976	710	1194
Average BNP before/after Hyponatremia (pg/ml)	79/1,094	97/1311	92/998	87/1,325
Serum AVP before/after hyponatremia (pg/ml)	5.4/10.1	7.3/13.6	6.2/9.7	7.1/12.5
CVP before/after fluid restriction(mm Hg)	5–6/2–3	4–5/2–3	5–6/3–4	4–5/2–3
Change of serum sodium after administration of isotonic saline(mmol/l)	–1	–2	0	–2
Change of serum sodium after administration of hydrocortisone(mmol/l)	+6	+5	+3	+4
CVP after administration of isotonic saline and hydrocortisone(mm Hg)	11–12	10–11	9–10	10–11

Unfortunately, after 48 h, the 24-h urinary sodium excretion and serum sodium levels of the patients were not significantly improved, while the clinical signs of hypovolemia, including decreased blood pressure and increased cardiac frequency, were detected in case 2 and case 4.CVP monitoring at this time showed that all patients' CVP were significantly lower than normal. An attempted treatment of supplemental isotonic saline solution was immediately implemented, with the intention to further distinguish SIADH and CSWS (Verbalis et al., 2013). For each patient, the positive balance of fluids at 1,000 ± 250 ml/d was ensured; with stable blood pressure, cardiac frequency, and normal CVP, an additional infusion of 1,000 ml of 0.9% sodium chloride solution was provided. Except for case 3, for whom the serum sodium remained unchanged, the serum sodium values of the remaining decreased slightly, while the levels of urinary sodium excretion increased in all cases. Subsequently, 100 mg of hydrocortisone was added, with intravenous infusion twice a day (Papadimitriou et al., 2007; Wu et al., 2016). This was expected to reduce the urinary sodium excretion while the supplemental saline was continued. The details of the sodium supplementation were based on the daily serum sodium values with relevant references (Adrogué and Madias, 2000; Verbalis et al., 2013). The increases in serum sodium and the decreases in urinary sodium excretion for all patients in early stages of treatment were obvious. However, even after 3 days of careful treatment, all patients' serum sodium values never fully returned to normal. The GCS of two patients began to decline slightly, and head computed tomography (CT) scans showed that the originally improved brain swelling situation had slight trend to become aggravated.

This sign was clearly a manifestation of excessive extracellular fluid; the CVP of all patients at this time were also significantly higher than normal (Table 2), indicating the hypervolemia status. Therefore, on the basis of the above-mentioned dual regimen, 20 mg furosemide was administered to the patients via intravenous injection twice a day (Soudan and Qunibi, 2012; Verbalis et al., 2013). After 1 day, the serum sodium level of case 3 returned to normal, and case 2 returned to normal after 36 h. The other two patients had also returned to normal within 48 h of the triple regimen.

### Follow-up and outcome

Since then, no hyponatremia has occurred during the application of the triple regimen. The patients left the ICU in 4–5 weeks after their injuries to start neurological rehabilitation treatment. At this time, only half doses of furosemide and hydrocortisone were administered. According to the total amount of urine sodium excretion, respectively, to give their 8–12 g of sodium chloride tablet per day (Kerns et al., 2014), and serum sodium was maintained at a normal level. The follow-up 3 months after injury showed that all patients were discharged and that the serum sodium levels, plasma osmolality and urine flow rate (ml/d) were in the normal range (Table 3) with a continuous sodium supplementation program, while the daily liquid intake was limited to no more than 1,000 ml (Furst et al., 2000). Hydrocortisone and furosemide had been discontinued since discharged. Urine specific gravity and 24-h total urine sodium were still slightly higher than the normal level. In addition to the disease itself, oral sodium supplement should also be part of the reason. Except for case 3, BNP value was still higher than the normal, which seemed to indicate that the pathological of CSWS was still in existence. AVP value was still at a high level, it seemed even higher than the initial stage. But at this time the hypoosmolality had been completely corrected, so the basal secretion level of AVP was bound to increase (Bankir, 2001).

**Table 3:** Laboratory data of patients after 3 months of traumatic brain injury.

**Parameters**	**Case 1**	**Case 2**	**Case 3**	**Case 4**
Serum sodium (mmol/l)	141	139	146	152
Urine flow rate (ml/d)	2,140	2,310	2,070	2,200
Urine specific gravity	1.039	1.037	1.033	1.041
Urine sodium excretion (mmol/24 h)	427	502	221	353
BNP (pg/ml)	413	667	95	322
AVP (pg/ml)	17.9	22.1	12.7	20.4

In 3–4 months after TBI, case 1, 3, and 4 subsequently sought treatment due to complaints of thirst; the laboratory test result showed normal serum sodium, so the fluid restriction was stopped, and the sodium chloride consumption was reduced by half. Before the 6-month follow-up after injury, fluid restriction, and supplemental saline were stopped for all cases. The serum sodium, urine specific gravity and total urine sodium excretion were normal for all cases except case 2 (Table 4). Because CVP measurement is an invasive examination, outpatient implementation of the inspection there were many inconvenience factors, so the kins of case 2 ultimately did not approve. The Glasgow Outcome Scores (GOS) are shown in Table 1. No relevant symptoms and signs were identified in case 2, and his neurological imaging did not change significantly compared to the preceding. Recent studies (Zaino et al., 2013; Ayus et al., 2016) have revealed that even mild and asymptomatic hyponatremia can result in subtle neurologic impairment and bone demineralization, leading to falls and associated bone fractures in the elderly. Despite the absence of massive scientific support, the cornerstone of treatment of chronic hyponatremia has been restriction of water intake (Berl, 2013). So the total daily intake of water was limited to 500 ml less than the daily urinary volume no further interventions. The follow-up at 12 months after TBI revealed that the sodium was 136 mmol/l. Pertinent laboratory data are shown in Table 4. The water restriction was continued, without complaint of mouth dryness partly because of his consciousness. For case 3, it has been less than 12 months since the injury. All laboratory test result were normal for other patients.

**Table 4:** Laboratory data of case 2 after 6 and 12 months of traumatic brain injury.

**Point-in-time (Months)**	**Serum sodium (mmol/l)**	**Urine flow rate (ml/d)**	**Urine specific gravity**	**Urine sodium excretion**	**BNP (pg/ml)**	**AVP (pg/ml)**
6	131	2,350	1.036	237	113	9.3
12	136	1,910	1.027	221	87	9.6

## Discussion

Hyponatremia occurred 1–2 weeks after TBI, at this time, the peak period of brain edema had passed following timely and effective surgery and post-operative drug therapy, and mannitol had been discontinued. Based on the results of the laboratory tests, osmotic dehydration, hyperglycemia, and other diseases that may cause hyponatremia were completely excluded (Filippatos et al., 2016). In particular, the concentrations of serum potassium and serum calcium were normal before the onset. During ICU, with appropriate analgesia and sedation, no significant pain stimuli presented in the patients; during the entire treatment, ventilators were not used for mechanical ventilation. Accordingly, the possible interference from the above factors can be excluded (de Denus et al., 2004). Diuretics, glucocorticoids, lithium, and carbamazepine were not administered to any of the four patients before hyponatremia (Sachdeo et al., 2002; Kazama et al., 2007; Bhuvaneswar et al., 2009; Lange-Asschenfeldt et al., 2013).

As shown in Table 2, the 24-h urinary sodium excretion levels were significantly higher than the previous average, but the increase in urinary flow rate was not obvious, the maximum value of not more than 3,000 ml/d. The CVP value of four patients was completely normal in the early onset of hyponatremia, so SIADH secretion-induced hyponatremia seems to be considered as the primary diagnosis accordance with the relevant literature (Hannon et al., 2012; Cuesta et al., 2016). As for the characteristics of increased urinary sodium excretion, SIADH, and CSWS also showed increased urinary sodium excretion. Therefore, the CSWS characterized by volume depletion was temporarily excluded from the diagnosis. However, fluid restriction not only did not improve the serum sodium level, but resulted in a state of hypovolemia. Based on this evidence, the diagnosis of SIADH should be rejected, nevertheless, the results of the attempted treatment of supplemental isotonic saline solution also seem to negate CSWS.

**Figure 1:**
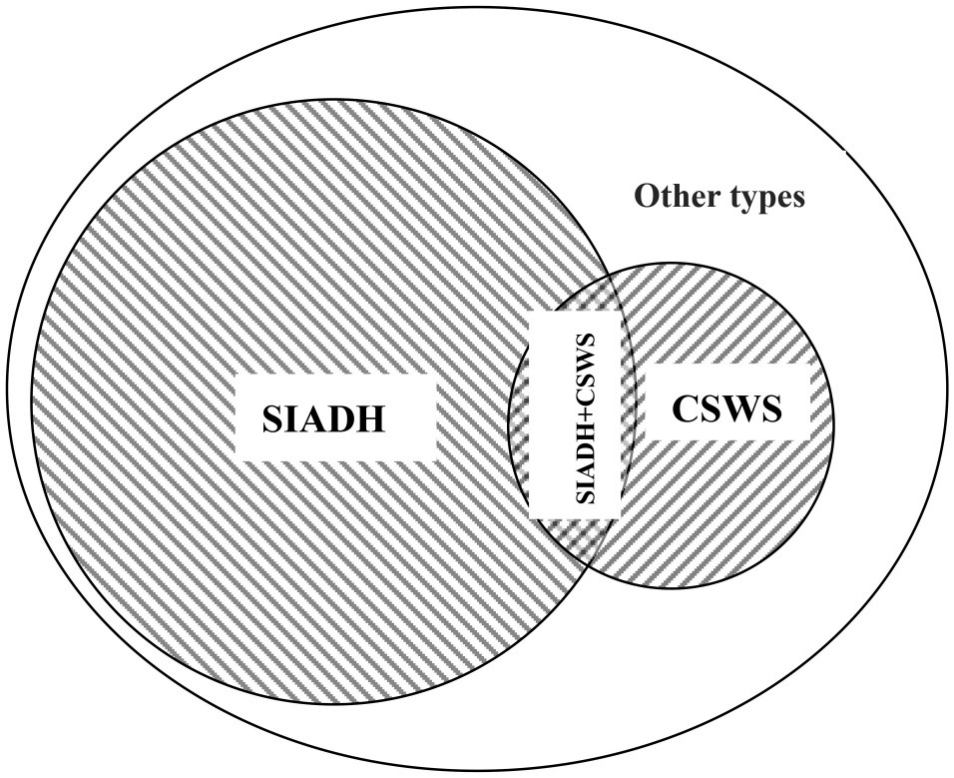
Etiology classification of hyponatremia after traumatic brain injury. SIADH, the syndrome of inappropriate antidiuretic hormone; CSWS, cerebral salt wasting syndrome; SIADH + CSWS, concurrence of inappropriate antidiuretic hormone secretion and cerebral salt wasting syndromes; Other types, other types of hyponatremia after traumatic brain injury, such as iatrogenic hyponatremia, adrenal insufficiency hyponatremia; Overlapping signs, serum Na^+^↓; Urine Na^+^↑; urine specific gravity ↑; urine flow rate (ml/d)—close to normal; CVP—completely normal.

Although the detection values of AVP and BNP can reveal largely the intrinsic pathological mechanism in most cases (Lu et al., 2008; Wu et al., 2016), in clinical practice, the cornerstone of the differential diagnosis of different types of central hyponatremia remains CVP testing. Nonetheless solely dependent on the CVP value for differential diagnosis, the result will be objective? All patients still maintained normal CVP values at the onset, so euvolemic hyponatremia was unquestionable. A typical SIADH should not show such a high urine specific gravity, and the 24-h total urine excretion is usually less. The above-mentioned details obviously did not support the diagnosis of SIADH; nevertheless, in the case of such a large amount of urinary sodium excretion, all patients could maintain a close to normal urine flow rate, which also showed that the ability of the kidney to reabsorb free water was very strong. In addition, for patients with sustained and significantly elevated AVP levels, the objective presence of SIADH must not be denied. Compared with the typical SIADH, the total amount of urinary sodium excretion in 24-h was obviously too extreme, which was clearly not caused by SIADH alone, but was most likely the result of multiple pathologic mechanisms acting simultaneously. In the face of such a large amount of urinary sodium excretion, even if the CVP value is completely normal, we cannot completely rule out the factual existence of CSWS. Although CSWS always occurs as a hypovolemic hyponatremia, but at the same time if there are excess free water reabsorption mediated by AVP far exceeding the physiological level, the volume depletion may not present. According to the literature (Wu et al., 2016), the vast majority of CSWS patients in the case of complete exclusion of heart failure, BNP detection values are significantly increased. Comprehensive analysis of the above-mentioned details, the pathomechanism of CSWS was likely to exist also. All these details further support that the pathology of SIADH and CSWS existed at the same time, and the exact etiology should be the concurrent syndrome of inappropriate antidiuretic hormone secretion and cerebral salt wasting syndrome (Figure 1).

Will this be the case: it was SIADH at the onset, and then converted to CSWS after treatment? Really, some believe that part of cases of CSWS is a consequence of misdiagnosing and inappropriate treatment of SIADH because treatment with mass isotonic salt solution may worsen diuresis and natriuresis (Hannon et al., 2012). In fact, all patients received fluid restriction on the first time after the onset rather than mass isotonic saline solution. Or the exact opposite order? All patients with the normal CVP at the onset of hyponatremia, which could completely rule out the possibility of single CSWS (Hannon et al., 2012; Verbalis et al., 2013; Taylor et al., 2017). After the failure of fluid restriction, we also once considered that it may be a special type of CSWS with “escape,” but then the treatment according to the CSWS principles was also invalid. There is just an interesting mechanistic possibility that the CSWS occurred initially since the beginning and the SIADH coming later as an allostasis mechanism to compensate the brain insult (Kim and Joo, 2009). However, the response of AVP to hypovolemia is far less sensitive than osmolality and does not occur without concomitant decrease in blood pressure (Kim and Joo, 2009). After actively supplementing the salt solution, all patients had returned to the normal volume status, and BNP value and urinary sodium excretion was still very high, all this clearly indicated that CSWS also existed except for SIADH (Lu et al., 2008; Wu et al., 2016).

On these basis, we thought that after continuous supplementation with saline solution and the administration of corticosteroid, the total amount of sodium in the patient's extracellular fluid was likely to reach a normal level. However, the inappropriate secretion of antidiuretic hormone caused the excessive absorption of water by the kidney resulted in dilutional hyponatremia and a hypervolemia status. Thus, for this particular type of euvolemic hyponatremia, the first thing to do is to actively supplement saline solution, while the application of corticosteroid to reduce urinary sodium excretion, diuretics are administrated to discharge excessive free water only when the CVP is slightly higher than normal, which may be the effective treatment for this complex, refractory hyponatremia. It was indeed a puzzling question whether SIADH and CSWS's pathophysiological processes are fully synchronized at the onset, and these cases were clearly difficult to provide too much of this. But at least one thing was certain, that was, in the whole course of disease, these two completely distinct pathophysiological state were meanwhile present *in vivo*. In these cases, case 2 was the most necessary to be discussed separately, whose follow-up data fully explained that CSWS is invariably self-limiting (Cerda'-Esteve et al., 2008) but chronic SIADH can often be seen in clinical practice (Chang et al., 2008; Dick et al., 2015).

## Concluding remarks

The contradiction of the early clinical signs of hyponatremia and the laboratory test parameters is precisely the result of the joint effects of these two pathological mechanisms. In the CSWS pathological process, the renal reabsorption of free water secondary to the increase in urinary sodium excretion was reduced, but the conditions of polyuria and hypovolemia were compensated by a large amount of free water reabsorbed by the renal collecting ducts in the pathological process of inappropriate antidiuretic hormone secretion. Thus, no clinical signs of hypovolemia and dehydration were observed, the 24-h total urine volume was far less than the typical CSWS level, and the CVP value could be maintained in a normal range. In the diagnosis of this special euvolemic hyponatremia, if the patient has a normal CVP value, the congestive heart failure, pain, hypotension, and other interference factors have been excluded, lab results showing the significantly and continuously elevated plasma AVP and BNP level, coupled with an extreme elevation of urine specific gravity and the failure of the attempted treatment of salt supplementation, can clearly suggest this syndrome. If the neurologist relies solely on CVP in the differential diagnosis, it is easy to fall into the trap of SIADH.

## Ethics statement

This study was carried out in accordance with the recommendations of the guidelines of the International Committee of Medical Journal Editors, the ethics committee of the Shanxi Provincial People's Hospital with written informed consent from all subjects. All subjects gave written informed consent in accordance with the Declaration of Helsinki. Taking into account the patients themselves had various degrees of disturbance of consciousness, the next of kin of all patients received an explanation of the research protocol and gave written informed consent.

## Author contributions

BS conceived of the study, and participated in its design and coordination, and draft the manuscript. LL participated in the design of the study, and contributed to revise the manuscript. TL carried out the collection and interpretation of data, took part in the data analysis and drafted the manuscript. BS also participated in the data analysis. All authors read and approved the final manuscript. All authors agree to be accountable for the content of the work.

### Conflict of interest statement

The authors declare that the research was conducted in the absence of any commercial or financial relationships that could be construed as a potential conflict of interest.
